# The rising cost of infective endocarditis in West Virginia

**DOI:** 10.1017/S0950268824001869

**Published:** 2024-12-26

**Authors:** Ruchi Bhandari, Noor Abdulhay, R. Constance Wiener, Dalton Smith, Melanie Fisher

**Affiliations:** 1 School of Public Health, West Virginia University, Morgantown, USA; 2 School of Dentistry, West Virginia University, Morgantown, USA; 3 School of Medicine, West Virginia University, Morgantown, USA

**Keywords:** electronic medical records, healthcare utilization, hospital charges, infective endocarditis

## Abstract

The financial burden of hospitalization from life-threatening infectious diseases on the U.S. healthcare system is substantial and continues to increase. The purpose of this study was to identify key predictors of high hospital charges for infective endocarditis at a major university-affiliated cardiac care centre in West Virginia.

A retrospective electronic medical records’ review was undertaken of all adult patients admitted for endocarditis between 2014–2018. Multiple linear regression analysis assessed the total charges billed to the patient account for their endocarditis hospitalization in the medical record.

Hospital charges have increased 12-fold during 2014–2018. Among the 486 patients, the median hospital charge was $198 678. About 47% of the patients underwent surgery incurring 70% of the total charges. Patients with hospital stays of ≥50 days accounted for a third of all charges. The multiple linear regression model accounted for 85% of the linear variance in the hospital charges. Median charges increased by 30.87% for patients with ≥9 consultations, 60.32% for those who died in the hospital, and 81.85% for those who underwent surgical intervention.

The study findings showed that complex care requiring multiple consultations, surgical interventions, and longer hospital stays were significantly associated with higher hospital charges for endocarditis treatment.

## Introduction

The United States (U.S.) has a reported infective endocarditis (IE) incidence of 15/100 000 which is considered the highest global IE rate [1]. Hospitalization rates for IE in the U.S. continue to increase, placing a significant financial burden on the healthcare system. Factors contributing to the escalating hospital charges for IE include the pre-disposing factor of injection drug use and intensive medical management requiring diagnostic tests, extended hospitalizations, long-term intensive antibiotic therapy, and surgical interventions.

The financial burden reflected in the total healthcare cost of IE-related hospitalizations has increased significantly from $1.58 billion in 2003 to $2.34 billion in 2016, with hospitalizations increasing from 34 488 to 54 405 per 100 000 during this period [2]. A significant factor driving the high cost of hospitalization for IE is the intensive management of the disease through antibiotics and/or surgery. While oral antibiotics can be effective, standard antibiotics can be compromised for some patients due to their inaccessibility of cardiac structures [3]. In these cases, surgery is the recommended course of action. Surgical treatment is a significant factor contributing to overall hospital charges for patients with IE. The total charges for hospitalizations involving surgery for 22 825 IE patients in North Carolina between 2007 and 2017 exceeded $400 million [4]. Specifically, the median charges for drug use associated-IE hospitalizations requiring surgery were significantly higher at $250 994 compared to $198 764 for non-drug use associated--IE surgery hospitalizations [4]. The high cost for IE may also be attributed to extended length of stay in the hospital to allow healthcare professionals to monitor and manage any post-operative complications, rehabilitation, and follow-up treatment [4–6].

Another important factor changing the epidemiology of IE in the past decade has been injection drug use, frequently requiring higher resource utilization and multidisciplinary care [7] resulting in substantially higher healthcare costs [8]. Results from a study using a nationally representative sample of U.S. patients revealed that the total inpatient hospital charges for infections related to opioid abuse, including IE, increased over three-folds from $190 million in 2002 to $700 million in 2012 [9]. Statewide and single-centre studies demonstrate hospitalization costs for patients with drug use associated-IE were twice as high in comparison to non-drug use associated-IE patients. For example, the median hospital charges for drug use associated-IE hospitalizations were $47 899 compared to $26 460 for non-drug use associated-IE hospitalizations in a single- centre study conducted during 2000–2016 [8]. Another statewide study also showed this contrast with median charges for drug use associated--IE hospitalizations at $60 333 vs. $34 968 for non-drug use associated-IE hospitalizations between 2007 and 2017 [4].

The higher readmissions of IE patients have been another significant source of elevated hospital costs. In a study utilizing the 2017 national readmissions database, 23% of all 56 357 hospitalizations for IE were readmitted within 30 days after their initial discharge [10]. The median hospitalization cost for patients during their initial admission was $20 241 [10]. However, when IE patients were readmitted, the median hospitalization cost increased to $22 059, indicating that the financial burden was much higher on readmission than initial admission [10].

Additionally, living in rural areas has been shown to be a predictor of poorer overall health outcomes and higher medical costs [11–12]. Admissions for IE in West Virginia (WV) experienced a significant increase of 456% between 2014 and 2018 [7]. A single- centre study in southern WV recorded 462 cases of IE between 2008 and 2015, resulting in hospital charges exceeding $17 million [12]. On average, a total of $70 000 was spent per patient with IDU-IE but only 22% ($3 829 701) were collected [12]. Another WV study examining IE patients who underwent tricuspid valve operations from 2012 to 2018 at West Virginia University Medical Centre showed that drug use associated-IE patients incurred significantly higher charges (totalling $291 037) compared to non-drug use associated-IE patients (totalling $235 620) [13]. Additionally, procedures associated with drug use had lower median reimbursement, at $42 063, compared to those not associated with drug use, at $70 393 [13].

The WV healthcare system continues to sustain losses of over $8 billion in gross domestic product annually due to the ongoing opioid epidemic [14, 15]. The high cost of hospitalization highlights the significant financial burden placed on patients and healthcare systems. The purpose of this study is to examine the healthcare burden measured as hospital charges and associated factors for patients with IE in the largest hospital in WV.

## Methods

### Study design, data sources, and variables

A retrospective review was conducted manually of electronic medical records (EMR) for all patients aged 18 to <90 years, who had their index admission for IE at a major university-affiliated hospital in WV between 1 January 2014 and 31 December 2018. Patients with IE were initially identified using the International Classification of Diseases, Tenth Revision, Clinical Modification (ICD-10-CM) codes and confirmed by detailed chart reviews. The data were abstracted for each patient from several sources within the EMR, such as laboratory tests, imaging results, note-taking on patient history, physical examination, consultations, surgeries, hospital stays, and discharge summaries. The number of subsequent admissions was recorded to obtain data on the frequency of readmissions during the study period. Information collected from EMR was entered into a HIPAA-compliant, secure dataset using the Research Electronic Data Capture (REDCap) system [16].

The outcome variable was hospital charges defined as total charges billed to the patient account for their IE hospitalization corresponding to their admission and discharge dates in the EMR. Hospital charges were explained by characteristics related to the year of admission, demographics, substance use, clinical features, and hospital utilization. *Demographic characteristics were classified by* sex (male and female) and age (18–44; 45–64; *>*64 years). *Substance use* was described by smoking status (current smoker, ex-smoker, and non-smoker), alcohol use (current alcohol use, prior alcohol use, and no alcohol use), history of drug use (yes/no), and type of drug used. *Clinical characteristics were explained by* comorbidities, number of comorbidities, psychiatric disorders, causative organism, and type of IE (tricuspid, mitral, aortic, and pulmonic). Lastly, *hospital utilization was broken down by* consultations received; surgery for IE (yes/no), length of hospital stay, length of ICU stay, discharge status (alive, against medical advice, and death), and readmission during the study period.

### Statistical analyses

Descriptive statistics was performed on categorical variables as counts and percentages. Continuous variables were presented as median and interquartile range (IQR). The primary outcome variable, hospital charges, was a continuous variable, presented separately as total charge as well as average (median) charge per person and the interquartile range for each variable. The medians were compared using the nonparametric independent samples median test. Variables with significant associations were selected in the multiple regression model. Multiple linear regression analyses were conducted to examine the association between the key dependent variable, hospital charges, and key predictor variables/potential confounders: age (continuous), history of drug use (yes/no), length of stay (continuous), surgery (yes/no), and number of consultations (0–5, 6–8, ≥9).

Assumptions meeting multiple linear regression were tested. These tests included sample size appropriateness, linearity, multicollinearity, normality of residuals, and testing for regression outliers. The initial study sample had 492 patients. Regression outliers were tested using leverage plots, studentized residuals, and Cook’s D. The five lowest observations and one highest observation in the residuals were identified as outliers and excluded from the analyses. Therefore, this study had 486 patients. The data normality of residuals was tested using the P–P plot. Natural log transformation was applied to the variable ‘total hospital charges’. The relationship between the length of hospital stay and hospital charges was curvilinear. Hence, to improve the linearity between the dependent and independent variables, and to boost the validity of statistical analyses, natural log transformation was also applied to the variable length of hospital stay. The multicollinearity assumption was tested using the Variance Inflation Factor. The predictor variables of interest were less than 5. All assumptions to use a multiple linear regression analysis were met in the model.

The final model included the dependent variable, total hospital charges, with the following variables that were significant in the bivariate association (age, alcohol use, length of hospital stay, surgery, substance abuse, number of drugs, and psychiatric disorders). Omnibus model fit was addressed with regression F test and an alpha of ≤0.05 was utilized. Adjusted R Square was used to test the effect size of the overall final model. Unstandardized and standardized beta coefficients with standard error were calculated for the multiple regression analyses. The relative effect of each factor on hospital charges was assessed by calculating: (a) the percent change for categorical variables compared to the reference category; or (b) per x units of a continuous variable (e.g. age in years) or log-transformed units of hospital length of stay. The percent change was calculated using the following formula: Percent Change = (exp (beta-k × units-k) − 1) × 100 where beta-k is the regression coefficient estimate and units-k are the units of measure for the regression coefficient k. The percent change for categorical variables was evaluated relative to the reference category and was interpreted alongside the intercept reference. Statistical significance was considered when the 95% confidence interval (CI) did not contain 0. All statistical analyses were conducted using SPSS software, version 28.0.

## Results

**Figure 1.**
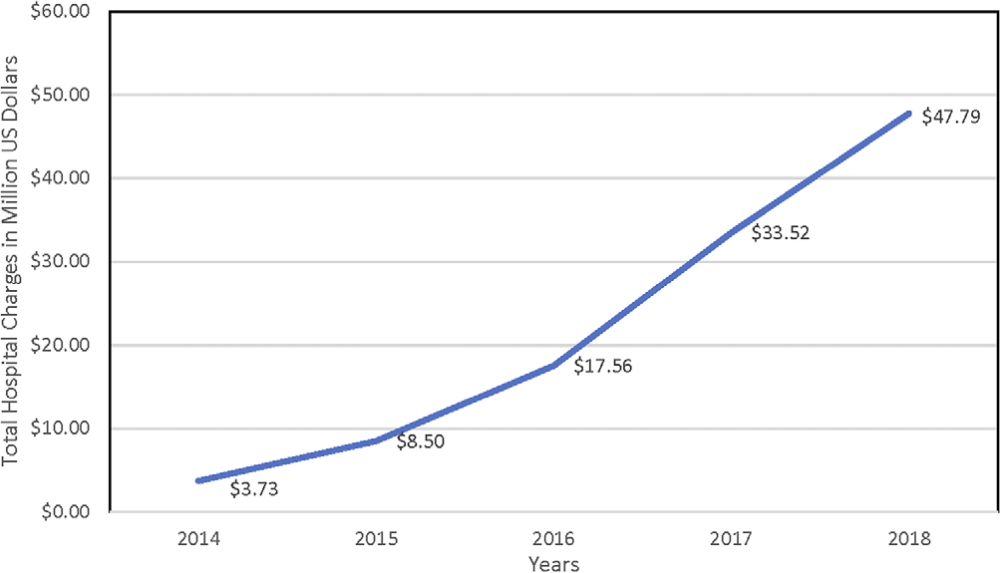
Total hospital charges (in Million U.S. Dollars).

The data were analyzed from 486 patients with IE who were admitted between, 1 January 2014 and 31 December 2018. The overall sum of total charges for all participants was $112 million (Median $198 678, IQR $213 309). Figure 1 represents the trend of total charges over the years, revealing a consistent increase in IE expenses over time. Baseline patient characteristics are presented in Table 1. From 2014 to 2018, IE admissions increased from 28 to 179. Healthcare charges rose from $3.7 million in 2014 to $48 million in 2018, although inflation only increased by 1.1% (0.8% in 2014–1.9% in 2018). While the number of patients increased 5.4 times, the associated hospital charges increased 12-fold over the study period.

**Table 1.** Baseline characteristics

Variables	N	%	Total charges (million U.S. $)	%	Median charge (U.S. $)	IQR^a^	p-value^b^
	**486**		**111.96**		**198 678**	**213 309**	
**Years**							**<0.001**
2014	28	5.76	3.73	3.33	81 965	83 380	
2015	55	11.32	8.50	7.59	124 445	150 198	
2016	95	19.55	17.56	15.69	143 771	202 141	
2017	126	25.93	33.52	29.94	236 640	213 371	
2018	179	36.83	47.79	42.69	244 632	199 249	
Missing	3	0.62	0.85	0.76			
**Sex**							0.785
Male	252	51.85	59.73	53.35	203 224	215 498	
Female	234	48.15	52.23	46.65	194 744	218 772	
**Age**							**<0.001**
18–44	300	61.73	71.35	63.73	224 960	219 777	
45–64	116	23.87	28.52	25.47	193 468	170 308	
65+	70	14.40	12.09	10.79	111 541	148 763	
**Smoking status**							**<0.001**
Current smoker	321	66.05	75.28	67.24	220 712	202 469	
Former smoker	91	18.72	23.56	21.05	194 093	262 652	
Non-smoker	63	12.96	10.96	9.79	125 024	169 560	
Missing	11	2.26	2.15	1.92			
**History of drug use**							**<0.001**
No	144	29.63	26.40	23.58	142 086	176 502	
Yes	341	70.16	85.55	76.41	233 175	209 710	
**Type of drug**							
Opiates^c^	288	59.26	74.34	66.40	238 196	213 175	**0.002**
Amphetamines	154	31.69	38.54	34.42	240 730	167 613	**<0.001**
Cannabinoids (Marijuana)	161	33.13	44.13	39.42	238 364	202 900	**<0.001**
Buprenorphine	137	28.19	34.14	30.50	238 530	195 103	**0.002**
Cocaine metabolites	125	25.72	35.39	31.61	255 694	215 186	**0.007**
Benzodiazepines	81	16.67	21.58	19.27	255 043	164 634	**<0.001**
Methadone	28	5.76	7.29	6.51	239 094	212 906	0.33
**Alcohol use**							0.103
Current alcohol use	126	25.93	32.02	28.60	229 060	245 664	
Former alcohol use	58	11.93	14.60	13.04	221 575	239 894	
No alcohol use	279	57.41	59.73	53.35	185 022	211 721	
Missing	23	4.73	5.61	5.01			

*Note:* Charges for the variable ‘Type of Drug’ exceed 100% because drug categories are not mutually exclusive.

a Interquartile Range.

b Values in bold indicate statistical significance.

c Opiates include Fentanyl, Carfentanyl, Heroin, Codeine, Morphine, and Oxycodone.

There was a similar distribution of male and female patients with IE. Participants aged 18–44 years comprised almost two-thirds (62%) of the total patients and hospital charges. Additionally, over two-thirds (66%) of patients were current smokers, representing a majority of total charges among this subgroup. An even higher proportion (70%) of patients had a history of drug use, and they incurred over three-quarters of the hospital charges ($85.5 million). Table 1 also presents the types of drugs used by participants prior to their hospital admission. Among these patients, 59% had used opiates, including fentanyl, carfentanyl, heroin, codeine, morphine, and oxycodone, accounting for healthcare charges totalling $74 million.

The clinical and surgical characteristics of the participants are presented in Table 2. Comorbidities played a significant role in the financial burdens associated with IE. The presence of multiple comorbidities in individuals corresponded to a greater increase in overall charges. Over 58% of the patients had some form of psychiatric disorder, which incurred hospital charges amounting to $70 million. Of the patients with psychiatric disorders, 44.6% had substance use problems. About 42% of the patients had tricuspid valve-affected IE incurring the most charges, followed by mitral valve-affected IE (34%). Among the causative organisms, *methicillin-resistant Staphylococcus aureus* (MRSA) associated IE incurred the highest charges, followed by *methicillin sensitive S. aureus* (MSSA)-associated IE.

**Table 2.** Clinical characteristics

Variables	N	%	Total charges (million U.S. $)	%	Median charges (U.S. $)	IQR^b^	p-value^b^
**Comorbidities**							
Psychiatric disorders	283	58.23	69.99	62.51	234 274	207 683	**<0.001**
Hypertension	176	36.21	36.94	32.99	170 148	221 022	**0.006**
Type 2 diabetes	89	18.31	18.33	16.37	143 391	214 666	**0.01**
Coronary artery disease	75	15.43	14.68	13.11	164 300	166 673	**0.003**
Chronic lung disease	61	12.55	13.62	12.17	171 757	247 441	**0.1**
Hyperlipidaemia	70	14.40	14.29	12.77	155 635	250 198	**0.014**
Acute kidney injury	30	6.17	8.32	7.43	213 791	225 546	**0.85**
Chronic kidney disease	48	9.88	11.52	10.29	141 966	308 106	**0.048**
Stroke	40	8.23	7.70	6.88	162 733	147 645	**0.013**
Peripheral vascular disease	41	8.44	9.59	8.56	163 877	272 153	0.192
Metastatic infections	19	3.91	3.66	3.27	152 575	181 958	**0.16**
Cancer	32	6.58	6.99	6.24	138 150	179 597	**0.1**
**Number of comorbidities**							**0.002**
0	54	11.11	12.25	10.94	206 343	216 537	
1	204	41.98	50.52	45.13	235 495	203 476	
2	90	18.52	18.76	16.75	168 856	200 225	
≥3	138	28.40	30,43	27.18	164 088	231 874	
**Psychiatric disorders**							
Substance use disorder	217	44.65	57.08	50.99	255 043	200 699	**0.001**
Depression	140	28.81	35.20	31.44	236 640	205 708	0.057
Anxiety	128	26.34	34.36	30.69	256 714	207 572	**<0.001**
Bipolar disorder	31	6.38	7.54	6.73	241 159	273 869	0.458
Post-traumatic stress disorder	35	7.20	8.34	7.45	232 231	181 286	0.483
**Causative organisms**							
MRSA – *Staphylococcus aureus*, Methicillin resistant	154	31.69	38.93	34.77	229 621	211 529	0.205
MSSA – *Staphylococcus aureus*, Methicillin sensitive	140	28.81	33.86	30.24	233 017	221 792	**0.021**
Other streptococci species	64	13.17	11.69	10.44	180 352	171 561	0.687
Enterococcus species	53	10.91	12.04	10.75	171 537	228 549	0.244
Candida species	43	8.85	14.47	12.92	289 260	321 810	**0.025**
Serratia species	33	6.79	8.39	7.49	237 029	212 545	0.279
Culture negative	18	3.70	3.40	3.04	143 605	200 264	0.23
Viridans streptococci	17	3.50	4.38	3.91	194 752	230 491	1
Klebsiella species	12	2.47	4.31	3.85	293 911	456 098	0.381
Other	53	10.91	11.42	10.20	171 170	207 554	0.244
**Type of infective endocarditis**							
Tricuspid	206	42.39	51.91	46.36	236 445	207 219	**<0.001**
Mitral	167	34.36	42.00	37.51	216 503	1,691 592	0.849
Aortic	136	27.98	34.20	30.55	189 211	242 329	0.613
Pulmonic	12	2.47	3.07	2.74	228 715	249 104	0.77

*Note:* Total Charges do not add to 100% for variables where categories are not mutually exclusive.

a Interquartile range.

b Values in bold indicate statistical significance.

Hospital utilization and associated charges are presented in Table 3. Infectious disease consultations incurred the highest hospital charges, followed by cardiac surgery, social work, physical and occupational therapy, and cardiology and psychiatry consultations. About 47% of the patients underwent surgery incurring 70% of the total charges. The longer length of hospital stays caused a greater increase in overall total charges. Patients with hospital stays of 50 days or longer accounted for a third of all charges totalling $37 million. Over one-third of the patients (36.6%) were hospitalized for 40 or more days. The majority of the patients (79%) were discharged alive, accounting for charges of $88 million.

**Table 3.** Hospital utilization characteristics

Variables	N	%	Total charges (million U.S. $)	%	Median charges (U.S. $)	IQR^a^	p-value^b^
**Consultations**							
Infectious disease	470	96.71	110.47	98.67	206 272	212 335	**0.005**
Cardiac surgery	394	81.07	100.82	90.06	234 282	209 320	**<0.001**
Cardiology	290	59.67	72.71	64.95	232 052	232 314	**0.007**
Social work	376	77.37	93.32	83.35	227 710	208 224	**<0.001**
Physical/Occupational therapy	315	64.81	81.54	72.83	231 873	226 035	**0.004**
Psychiatry	253	52.06	71.02	63.43	265 911	185 859	**<0.001**
Nephrology	167	34.36	50.55	45.15	267 699	268 354	**<0.001**
Dentistry	185	38.07	52.87	47.22	252 120	181 415	**<0.001**
Spiritual counselling	173	35.60	49.64	44.34	251 728	235 401	**<0.001**
General surgery	129	26.54	41.48	37.05	277 482	271 633	**<0.001**
Neurology	112	23.05	2.94	2.63	228 589	236 239	0.914
Vascular	108	22.22	31.10	27.78	238 678	256 873	0.102
Orthopaedic surgery	99	20.37	26.63	23.79	231 873	253 860	**0.043**
Pain management	73	15.02	23.83	21.29	272 717	150 296	**<0.001**
Neurosurgery	68	13.99	17.59	15.71	234 788	183 512	0.896
Interventional radiology	68	13.99	25.27	22.57	343 688	244 928	**<0.001**
Ophthalmology	65	13.37	15.99	14.28	238 364	172 933	0.286
Individual therapy	16	3.29	4.90	4.38	293 426	123 429	**0.022**
**Number of consultations**							
1–5	155	31.89	19.82	17.71	89 865	133 585	**<0.001**
6–8	204	41.98	46.02	41.11	211 503	178 893	
≥9	127	26.13	46.11	41.19	321 965	189 610	
**Surgery**							**<0.001**
No	256	52.67	33.62	30.03	102 553	117 838	
Yes	230	47.33	78.34	69.97	296 800	166 053	
**Length of hospital stay (days)**							
≤4	30	6.17	1.02	0.91	29 623	16 391	**<0.001**
5–9	56	11.52	3.84	3.43	48 443	26 702	
10–19	99	20.37	13.03	11.64	119 175	97 959	
20–29	75	15.43	18.01	16.09	220 662	128 862	
30–39	48	9.88	11.53	10.29	237 494	136 829	
40–49	97	19.96	27.09	24.20	269 645	158 190	
≥50	81	16.67	37.44	33.44	394 035	203 820	
**Discharge status**							**0.015**
Discharge alive	367	75.51	88.40	78.96	217 166	218 828	
Discharge against medical advice	74	15.23	11.78	10.52	134 005	203 061	
Death	45	9.26	11.78	10.52	187 158	207 379	
**Readmission**							
Yes	402	82.72	18.23	16.28	216 835	179 118	0.549
No	84	17.28	93.73	83.72	196 267	225 804	

*Note:* Total Charges do not add to 100% for variables where categories are not mutually exclusive.

a Interquartile range.

b Values in bold indicate statistical significance.

The multiple linear regression model accounted for 85% of the linear variance in the dependent variable (natural log of total hospital charges) adjusting for model complexity (Adjusted R^2^ = 0.849), indicating a large association between the predictor variables selected in the model (Table 4). In the adjusted model, the percent increases in median charges for consultations were as follows:1–5 consultations, $89 865; 6–8 consultations, $211 503 (11.18% increase); and ≥ 9 consultations, $321 965 (30.87% increase). Compared to patients who were discharged home, there was a 60.32% increase in the median charges for patients who died in the hospital. Similarly, relative to patients treated by medical management alone, patients who had surgical intervention incurred an 81.85% increase in median charges (beta coefficient 0.598, 95% CI 0.528–0.669). Within the full model, a statistically significant association was observed between total hospital charges and length of hospital stay (beta coefficient 0.662, 95% CI 0.617–0.708).

**Table 4.** Multiple linear regression of hospital charges

	B#	Standard error	β##	t	p-value^a^	95% Confidence interval		% Change
						Lower	Upper	
Constant	9.29	0.12		76.98	**<0.001**	9.057	9.531	
Surgery	0.60	0.04	0.34	16.59	**<0.001**	0.528	0.669	81.85
In-hospital death	0.47	0.06	0.16	8.34	**<0.001**	0.361	0.584	60.32
Length of stay (log-transformed)	0.66	0.02	0.67	28.44	**<0.001**	0.617	0.708	15.92
Consultations 6–8	0.11	0.04	0.06	2.68	**0.008**	0.028	0.183	11.18
Consultations ≥ 9	0.27	0.05	0.14	5.63	**<0.001**	0.175	0.363	30.87
Discharge against medical advice	0.01	0.05	0.00	0.15	0.877	−0.086	0.101	0.70
Age	0.00	0.00	0.07	2.54	**0.011**	0.001	0.007	0.40
Current/former smoker	0.01	0.05	0.00	0.19	0.85	−0.091	0.11	1.01
Comorbidities	0.03	0.04	0.02	0.76	0.448	−0.052	0.117	3.36
Drug use	0.01	0.05	0.01	0.23	0.819	−0.091	0.115	1.21

Note: Dependent Variable: Hospital charges is natural log transformed. Adjusted R Square: 0.849. B#: Unstandardized Beta Coefficients. β##: Standardized Beta Coefficients.

a Values in bold indicate statistical significance.

## Discussion

Results from this study provide valuable insights into the evolving landscape of healthcare charges amounting to $112 million associated with IE admissions over a 5-year period. In this study, the admissions at one hospital system with a catch basin of the state of WV rose from 1.56 admissions/100 000 population in 2014 to 9.94/100 000 in 2018. Additionally, the median charge per IE patient in the WV hospital system grew from $81 965 per patient in 2014 to triple that amount ($244,632) in 2018. With a minor rise in inflation during that period (0.8% in 2014 and 1.9% in 2018), this is an extreme rise in patient management charges. The hospital charges in West Virginia for patients with IE are substantially higher than the national estimates. At the national level, the mean inflation-adjusted cost per admission decreased from $45 810 in 2003 to $43 020 in 2016 [2].

The demographic and clinical characteristics of IE patients shed light on the factors driving these escalating costs. Surgery significantly contributed to the rising financial challenges associated with IE in our study. Nationally, IE surgeries increased by 1.7-fold during 2011–2018 [17]; however, there was a 26-fold increase in WV during 2014–2018 [18]. Patients undergoing surgical treatment for IE require pre-operative and post-operative hospital care [18, 19], which adds significantly to the overall charges. Furthermore, patients admitted to the ICU following surgery further escalate these costs [20]. Patients undergoing surgical treatment for IE require an increased demand for hospital resources, which also contributes to the overall cost [18, 20]. In a study conducted among 369 patients who underwent surgical intervention for IE, the financial burden on the healthcare system was the median charge of $60 072 per patient [21]. Patients who undergo surgical procedures during hospital admissions typically experience longer hospital stays compared to those who do not have surgery [4, 8, 18, 22, 23].

Patients who had multiple comorbidities contributed to a greater increase in overall healthcare costs. Having multiple medical conditions can lead to increased use of healthcare services and higher associated costs [24]. Treatment for individuals with multiple comorbidities requires tailored care that may extend over a longer duration [25–27], resulting in prolonged hospital stays, and increased overall charges [21].

A notable proportion of IE patients had a history of drug use, representing a majority of the total healthcare costs. While nationally, the prevalence of substance use among patients hospitalized for IE was 21% during 2015–2019 [28], the prevalence was substantially higher for WV patients [7]. Treating IE in individuals who use drugs requires extensive and costly multidisciplinary care [29]. A previous study found that in 2015, patients with drug use-associated IE accounted for a total of $9.3 million in healthcare costs [30]. The cost of hospitalizations for cases of IE that are associated with opioid use doubled between 2003 and 2016 [11]. The amount of money spent on hospitalizations for opioid-associated infections has increased at a much faster rate than the actual increase in the number of individuals being hospitalized for these issues [9]. Even after accounting for inflation, the rise in costs remains high relative to the increase in hospitalizations [9]. Factors such as escalating treatment expenses, greater resource use, and increased severity of illness all contribute to these elevated costs [9].

In this study, MRSA-associated IE incurred the highest charges. MRSA infections can be serious, and there are significant costs associated with treating these infections. In a study on patients hospitalized in the Department of Veterans Affairs healthcare system in the U.S., MRSA was the pathogen with the highest aggregate cost of $1.2 billion for community-onset infections and $580.2 million for hospital-onset infections in 2017 [31]. The authors concluded that MRSA infections had the highest total aggregate cost nationally because of their heavy financial burden.

Lastly, specific geographic and cultural aspects of the rural landscape add a burden to healthcare costs. Researchers have frequently concluded that rural populations are more likely to be poor, lack health insurance, engage in risky behaviours, and be sicker compared to their urban counterparts [32, 33]. Patients in rural areas have to travel greater distances to access healthcare, which is costly and requires time away from work. In addition, rural topography and transportation challenges add further barriers to accessing healthcare centres. Rural populations also face decreased access to home health or psychiatric resources and stigma regarding drug use [34]. Studies from other rural Appalachian states also indicate a similar trend in hospitalization due to IE [35]. Since these states face more financial challenges, the burden added by the steeply rising IE hospitalizations is more significant.

### Strengths and limitations

This study is strengthened by the exhaustive search of EMR from the premier hospital in WV that treats IE. Unlike most studies that have relied solely on ICD codes, our study further validated the initial IE diagnosis based on ICD codes with several clinical indicators retrieved from a comprehensive manual medical chart review of each patient. In addition, the manual chart review provided validated information on clinical and substance use-associated characteristics. Another strength of the study is that we analyzed data for individual patients and not hospitalizations. We were also able to abstract long-term data, such as the readmissions of the patients, although we did not have information on patient outcomes such as out-of-hospital mortality or subsequent admissions to other hospitals. Our study is subject to other limitations. It is a single-centre study, although the centre is the largest cardiac care centre in WV. In addition, the results are dependent on the data available in the EMR. Information was limited on socio-demographic variables, such as education, income, and duration of drug use. There was a potential for drug use data to be misclassified if patients had not disclosed previous drug use upon hospital admission. This bias was also a study limitation.

## Conclusion

In conclusion, hospital charges related to IE continue to have a significant financial burden on the healthcare system. Many patients with IE in WV required complex care and experienced significant morbidity. Prolonged length of hospital stays, surgeries, readmissions, and other medical consultations and procedures resulted in high hospital charges. The authors believe that interventions need to focus on early diagnosis and treatment of patients with IE to reduce the need for complex care, complications, extended hospitalizations, and the associated high hospital charges.

## Data Availability

Data underlying this study are from an approved repository that houses clinical data from the healthcare systems in West Virginia. These data contain full Protected Health Information (PHI) and thus, legally cannot be shared publicly. Patient data are available upon request from Wes Kimble (wkimble1@hsc.wvu.edu), Director of Research Data Analytics at the West Virginia Clinical and Translational Science Institute, for researchers who meet the criteria for access to confidential data. Hospital charges data were obtained from the Finance and Reimbursement Department at West Virginia University Health System.
